# Aphotic N_2_ Fixation in the Eastern Tropical South Pacific Ocean

**DOI:** 10.1371/journal.pone.0081265

**Published:** 2013-12-12

**Authors:** Sophie Bonnet, Julien Dekaezemacker, Kendra A. Turk-Kubo, Thierry Moutin, Robert M. Hamersley, Olivier Grosso, Jonathan P. Zehr, Douglas G. Capone

**Affiliations:** 1 Mediterranean Institute of Oceanography, Institut de Recherche pour le Développement, Aix Marseille Université, Centre National de la Recherche Scientifique Marseille/Noumea, New Caledonia, France; 2 Department of Ocean Sciences, University of California Santa Cruz, Santa Cruz, California, United States of America; 3 Environmental Studies, Soka University of America, Aliso Viejo, California, United States of America; 4 Department of Biological Sciences, University of Southern California, Los Angeles, California, United States of America; Uppsala University, Sweden

## Abstract

We examined rates of N_2_ fixation from the surface to 2000 m depth in the Eastern Tropical South Pacific (ETSP) during El Niño (2010) and La Niña (2011). Replicated vertical profiles performed under oxygen-free conditions show that N_2_ fixation takes place both in euphotic and aphotic waters, with rates reaching 155 to 509 µmol N m^−2^ d^−1^ in 2010 and 24±14 to 118±87 µmol N m^−2^ d^−1^ in 2011. In the aphotic layers, volumetric N_2_ fixation rates were relatively low (<1.00 nmol N L^−1^ d^−1^), but when integrated over the whole aphotic layer, they accounted for 87–90% of total rates (euphotic+aphotic) for the two cruises. Phylogenetic studies performed in microcosms experiments confirm the presence of diazotrophs in the deep waters of the Oxygen Minimum Zone (OMZ), which were comprised of non-cyanobacterial diazotrophs affiliated with *nifH* clusters 1K (predominantly comprised of α-proteobacteria), 1G (predominantly comprised of γ-proteobacteria), and 3 (sulfate reducing genera of the δ-proteobacteria and *Clostridium* spp., *Vibrio* spp.). Organic and inorganic nutrient addition bioassays revealed that amino acids significantly stimulated N_2_ fixation in the core of the OMZ at all stations tested and as did simple carbohydrates at stations located nearest the coast of Peru/Chile. The episodic supply of these substrates from upper layers are hypothesized to explain the observed variability of N_2_ fixation in the ETSP.

## Introduction

The efficiency of oceanic carbon (C) sequestration depends upon many factors, among which is the availability of nutrients to support phytoplankton growth in the illuminated surface ocean. In particular, large amounts of nitrogen (N) are required, as it is an essential component of proteins, nucleic acids and other cellular constituents. Dissolved N in the form of nitrate (NO_3_^−^) or ammonium (NH_4_^+^) is directly usable for growth, but concentrations of fixed N are low (<1 µmol L^−1^) and often growth-limiting in most of the open ocean euphotic zone [1]. Dinitrogen (N_2_) gas dissolved in seawater, on the other hand, is very abundant in the euphotic zone (ca. 450 µmol L^−1^) and could constitute a nearly inexhaustible N source for the marine biota. However, only certain prokaryotic ‘N_2_-fixers’ (or diazotrophs) are able to use this N source since they can break the triple bond between the two N atoms of the N_2_ molecule, and convert it into a usable form (i.e. NH_3_) for assimilation.

The focus of much recent marine N_2_ fixation research has been on the NO_3_^−^-poor environments of the surface tropical ocean, where it may sustain up to 50% of ‘new’ primary production [2], [3]. The filamentous cyanobacterium *Trichodesmium spp.*, which is widespread in the tropical ocean and has a macroscopic growth form [4], may fix from 60 [5] to 80 Tg of N per year [6]. Until the last decade, this organism was the focus of the bulk of research as it is conspicuous and easily collected [4]. However, since then, studies of the abundance and diversity of the *nifH* gene required for N_2_ fixation have elucidated the importance of unicellular pico- and nano-planktonic cyanobacteria [7], [8], extending the geographical extent of diazotrophy beyond tropical waters [9], and potentially narrowing the gap between direct measurements and geochemically-based global marine N fixation rates [5]. These molecular tools have also revealed the presence of putative non-cyanobacterial diazotrophs (possessing and potentially expressing the *nifH* gene) in diverse aquatic environments [10], including surface seawater, hydrothermal vents and lakes [11] and references therein). In marine waters, these diazotrophs seem to be almost ubiquitous [12], but few studies e.g. [13], [14] have focused on these non-cyanobacterial diazotrophs, and our knowledge of their distribution in the ocean and their biogeochemical importance for the marine N budget is still very limited.

The N budget for the global ocean is poorly constrained, with some suggestions that sinks (denitrification and anammox) exceed sources (N_2_ fixation) [15]. The high energy and iron (Fe) requirements [16], [17] of the N_2_ fixation reaction have implied that this process occurs mainly in the large oligotrophic areas of the ocean that are depleted in fixed N, and where fixing N_2_ gives an ecological advantage. This may be particularly the case in areas which receive high Fe-rich Saharan dust such as the North Atlantic [18], or which are under the influence of terrigenous and submarine Fe sources, such as the North Pacific near Hawaii [19], [20] or the South West Pacific [20], [21], [22], [23]. However, recent studies [24], [25] have hypothesized that N_2_ fixation might also be associated with denitrified surface waters over oxygen minimum zones (OMZs), which have measureable NO_3_^−^, but are depleted in N relative to phosphorus (P). This hypothesis has been recently confirmed in the coastal surface waters of the Peruvian-Chilean upwelling [26], [27] as well as throughout the eastern tropical South Pacific Ocean (ETSP) [28], where depth-integrated rates over the upper water column were comparable to those found in subtropical gyres. *nifH* sequences recovered from these areas within the upper 200 m of the ocean were mostly non-cyanobacterial and clustered with known heterotrophic sequences [26]. This led us to explore N_2_ fixation in the aphotic zone of the ETSP.

Previous studies conducted in surface waters of the ETSP indicated that N_2_ fixation was highly variable in space and time, with depth-integrated rates varying from 10- to 30-fold between cruises performed at the same locations [26], [28]. Although the activities of heterotrophic diazotrophs might potentially be contributing to this high temporal variability, very few studies have examined the regulation of N_2_ fixation by heterotrophic bacteria in marine waters. Organic C availability has been hypothesized to control marine heterotrophic N_2_ fixation [29] as a consequence of the high energy requirements of the reaction, but, to our knowledge, the effect of organic molecules on heterotrophic N_2_ fixation has never been studied in OMZs.

In this study, we investigated N_2_ fixation along a transect across the ETSP in 2010 and 2011 through temperature, oxygen and nutrient gradients. We quantified N_2_ fixation throughout the 0 to 2000 m depth range in order to evaluate its potential biogeochemical impact on the marine N budget, and we conducted aphotic nutrient addition bioassays in the core of the aphotic OMZ in order to investigate which nutrients might control N_2_ fixation in this environment. We also phylogenetically characterized the diazotrophs community composition in the core of the OMZ and how it responded to some of the nutrient amendments.

## Methods

**Figure 1:**
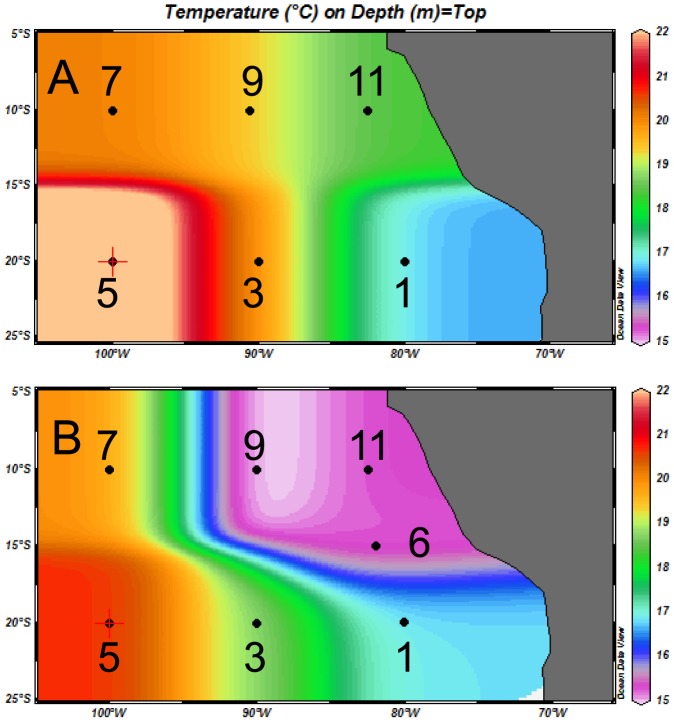
Location of stations superimposed upon seawater temperature at 75-m-depth. (A) the 2010 cruise (R/V Atlantis), and (B) the 2011 cruise (R/V Melville). Station numbering are identical to that of 2 companion papers [28], [42]).

Our research was carried out during two cruises in the ETSP, aboard the R/V Atlantis in February and March 2010, and the R/V Melville in March and April 2011. Experiments were performed along a transect that began in northern Chile and ran west along 20°S, from the nutrient-rich waters at 82°W to the more oligotrophic and low-NO_3_^−^ waters at 100°W, and returned along 10°S (Fig. 1). No specific permissions were required for these locations/activities as both cruises took place in international waters. This study did not involve endangered or protected species. The coastal waters of this region of the ETSP are characterized by a permanent wind-driven upwelling of cool nutrient-replete water (Fig. 1), which supports high primary productivity and a persistent subsurface OMZ, where O_2_ concentrations are low enough to induce the anaerobic processes of the N cycle, such as denitrification and anammox [30], [31], [32]. These O_2_-deficient waters are carried by Eckman transport westward beyond the limit of our transect. The ETSP is subjected to the inter-annual climactic variability of the El Niño-Southern Oscillation (ENSO), which modulates the strength of the upwelling. The 2010 cruise took place during an El Niño event (Multivariate ENSO index: 1.52) and the 2011 cruise during a La Niña event (Mutivariate ENSO index: −1.49) [NOAA Climate Diagnostics Center, Wolter and Timlin (1993, 1998); data from http://www.esrl.noaa.gov/psd/enso/mei.table.html].

### Hydrographic and nutrient measurements

Hydrographic and nutrient measurements were performed at 6 stations in 2010 and 7 stations in 2011 (Fig. 1). Vertical profiles of temperature, chlorophyll *a*, fluorescence and dissolved oxygen were obtained using a Seabird 911 plus CTD equipped with a model 43 oxygen sensor and a Wetlabs ECO-AFL/FL chlorophyll fluorometer. Oxygen values were calibrated by micro-Winkler [33]. Seawater samples were collected at selected depths using a rosette equipped with 24 12-L Niskin bottles. Samples for inorganic nutrient (NO_3_^−^ and PO_4_^3−^ concentrations) analyses were collected in acid-washed 20-mL plastic bottles. Nutrient concentrations were determined using standard colorimetric techniques [34] on a Bran Luebbe AA3 autoanalyzer. Detection limits for the procedures were 0.02 to 0.52 µmol L^−1^ for NO_x_ [nitrite (NO_2_^−^)+NO_3_^−^] and 0.005 to 0.083 µmol L^−1^ for PO_4_^3−^.

### Vertical profiles of N_2_ fixation

Rates of N_2_ fixation were measured using the ^15^N_2_ tracer method [35]. Water samples were dispensed into acid-leached 4.5-L polycarbonate bottles. During the 2010 cruise, this work was exploratory and unreplicated (except for nutrient addition bioassays, see below) measurements were made at 12 to 14 depths between the surface and 2000 m at stations 1, 9 and 11. During the 2011 cruise, samples were collected at stations 1, 5, 6, 7, 9 and 11 in triplicates at 12 depths between the surface and 2000 m, with a specific focus on O_2_ gradients. Depths were chosen in order to sample the oxycline, at least 3 depths within the core of the OMZ, as well as an additional 3 within the second increasing oxygen concentrations below the OMZ. Most of these depths were located in the aphotic zone.

On both cruises, specific care was taken to avoid O_2_ contamination and to perform incubations under strict oxygen-free conditions as described in [36]. Briefly, before each profile, the 36 4.5-L bottles were filled with deionized water, then the deionized water was flushed with argon and finally filled with seawater via tubing into the bottom of the argon-filled bottles to minimize gas exchange. Bottles were then closed with septa and spiked with 3 mL ^15^N_2_ (99 atom % EURISO-TOP) via a gas-tight syringe. Each bottle was shaken 30 times to fragment the ^15^N_2_ bubble and facilitate its dissolution. Recent work has suggested that with this method, there may be incomplete equilibration of the added ^15^N_2_ gas bubble with the seawater sample, resulting in a dissolved ^15^N_2_ concentration in the sample that is lower than the equilibrium value assumed in the calculation of ^15^N_2_ fixation rates [37]. This may lead to a potential underestimate of N_2_ fixation rates [38], [39]. Therefore, the values given in the present study should be considered as minimum estimates (discussed below). Bottles were then incubated either in on-deck incubators at irradiances specific from the sampling depth using blue screening and cooled with circulating surface seawater (photic samples), or in dark rooms at 12°C or 5°C depending of the sampling depth. After incubation, the triplicate bottles from each depth were filtered onto precombusted (4 h at 450°C) 25-mm GF/F filters. Filters were stored at −20°C until the end of the cruise, then dried for 24 h at 60°C and stored dry until mass spectrometric analysis. During the 2011 cruise, an extra 4.5-L bottle was collected at each depth of the profile, spiked with ^15^N_2_ and immediately filtered in order to determine the initial background δ^15^N in the particulate organic N (PON) for calculations of N_2_ fixation rates. During the 2010 cruise, the value of δ^15^N in air (0.00366) was used as a reference value for these calculations, which may introduce a potential bias, except at Station 1 where ^15^N atom % of the PON at depth was available.

### Nutrient addition bioassays in the core of the OMZ

Nutrient addition bioassays of N_2_ fixation were performed at one single depth in the core of the OMZ (based on O_2_-CTD profiles) at 3 stations (Stations 5, 7 and 11, between 140- and 450-m depth) during the 2010 cruise and at 6 stations (Stations 1, 5, 6, 7, 9 and 11, between 320- and 475-m depth-) during the 2011 cruise. All experiments were performed in triplicate and under strict oxygen-free conditions (using the argon flushing method described above) to avoid inhibition of N_2_ fixation by oxygen. Immediately after collection, bottles were capped with septa and amended with nutrients via syringes. During the 2010 cruise, at each of the 3 stations, triplicate bottles were left as unamended controls, and a second set of bottles was amended with glucose to obtain a final concentration of 10 µmol L^−1^. During the 2011 cruise, triplicate bottles were left as unamended controls, and a second set of triplicate bottles was amended with a mixture of three simple carbohydrate substrates (39% glucose, 29% acetate and 32% pyruvate, final total concentration of 1 µmol carbohydrate L-1) to test the effect of a source of dissolved organic C (DOC) on N_2_ fixation. A third set was amended with a mixture of three amino acids as a source of both DOC and dissolved organic N (DON) (20% leucine, 23% glutamic acid and 56% alanine) to reach a final concentration of 1 µmol amino acids L^−1^. The proportion of each carbohydrate and amino-acid has been chosen in order to add the same quantity of organic C in the two treatments (4 µmol L^−1^). A fourth set was amended with ATP (source of dissolved organic P, DOP) to reach a final concentration of 1 nmol L^−1^, and a fifth set was amended with 8 µmol L^−1^ of NO_3_^−^ to test its potential inhibitory effect on heterotrophic N_2_ fixation. Bottles were then incubated in a dark cold room at 12°C for 24 h in order to leave enough time to induce any potential nutrient stimulation. After 24 h, all bottles were spiked with ^15^N_2_ as described above, and incubation was continued under the same conditions for an additional 24 h. At the end of each incubation, the three treatments and control replicates were filtered as described above in order to measure N_2_ fixation rates, and amplification of the *nifH* gene (2010 only). Samples were also collected from bottles sacrificed at time zero in order to quantify background NO_x_ and PO_4_^3−^ concentrations at every station. NO_x_ concentrations were also measured just after the NO_3_^−^ additions in order to confirm the added concentrations at the beginning of the incubations (data not shown).

### Mass spectrometric analyses

The isotopic enrichment of particulate N after the incubation of seawater with ^15^N_2_ was measured by continuous flow isotope ratio mass spectrometry of pelletized filters (Europa Integra-CN), calibrated every 10 samples using reference material (International Atomic Energy Agency [AIEA], Analytical Quality Control Services). The linearity of ^15^N atom % as a function of increasing sample PON mass was verified as detailed in [40] on both natural and ^15^N enriched material. This step is critical in ultra-oligotrophic environments or deep waters, where suspended PON concentrations are low. ^15^N atom % was linear (Fisher test, p<0.01) between 0.20 and 39 µmol N, which is within the range of PON measured in all of our samples (0.27 to 4.91 µmole N depending on the station and depth).

Detection and quantification limits for particulate N were calculated daily, as 3 times and 10 times the standard deviation of ^15^N analysis of blanks, respectively. Detection limits ranged from 0.10 to 0.17 µmole N, and quantification limits ranged from 0.13 to 0.26 µmole N, depending on the station. The ^15^N isotope enrichment of a sample was calculated using the ^15^N atom % excess over the ^15^N atom % in samples taken from the same station at time zero, which was determined on bottles filtered immediately after adding ^15^N_2_. We considered the results to be significant when ^15^N excess enrichments were greater than 3 times the standard deviation obtained with ten AIEA references (^15^N atom % >0.0005). The quantification limit of N_2_ fixation in this study was 0.01 nmol L^−1^ d^−1^. If only one of the 3 replicate measurements was quantifiable, the average of the 3 replicates was forced equal to zero, in order to provide minimum estimates of N_2_ fixation.

In order to determine areal rates, N_2_ fixation measurements were trapezoidally depth-integrated from the summed products of the average of two adjacent rate measurements (including those equal to zero) with the depth interval between them. The standard deviation on the triplicates (2011 cruise) was also used for a trapezoidally depth-integration in order to obtain the standard deviation on integrated rates.

### Statistical analysis

Controls and experimental nutrient treatments were compared using a 2-tailed non parametric Mann-Whitney mean comparison test (n = 3, α = 0.05, unpaired samples).

### Phylogenetic characterization of diazotrophs

In order to characterize the potential diazotrophs present in the core of the OMZ that responded to the addition of glucose, nucleic acid samples were collected from triplicate bioassays during the 2010 cruise for amplification of the *nifH* gene. At T0 and at the termination of the experiment, bottles were immediately filtered as described in [41] onto 25-mm, 0.2-µm Supor filters (GE Osmotics, Minnetonka, MN), and immediately flash frozen in liquid N_2_. All filters were stored at −80°C thereafter.

DNA samples were extracted using the Qiagen All Prep kit (Valencia, CA), according to manufacturer's guidelines, with modifications to include freeze-thaw and bead-beating steps to disrupt the cells [42]. The wash steps of this protocol were automated using a QIAcube (Qiagen). DNA extracts were stored at −20°C until use.

Nested PCR amplification targeting a fragment of the *nifH* gene was carried out using degenerate primers *nifH*1-4 [43], [44] using the reaction and thermocycling conditions described in [42]. Amplicons were purified using a QIAquick Gel Extraction Kit (Qiagen) and cloned using the TOPO TA Cloning Kit for Sequencing (Invitrogen, Carlsbad, CA) according to the manufacturer's guidelines. Purified recombinant plasmids containing partial *nifH* sequences were recovered from clones using the Montage Plasmid Miniprep96 Kit (Millipore, Billerica, MA) and sequenced using Sanger technology at the UC Berkeley DNA Sequencing Center. All DNA extractions and as PCR preparations were performed in a PCR-amplicon free facility at UCSC described in [42].

Sequencher 5.1 sequence analysis software (Gene Codes Corporation, Ann Arbor, MI) was used to remove vector contamination and low-quality reads from raw sequences. All resulting partial *nifH* sequences were imported into a curated *nifH* database (http://pmc.ucsc.edu/~wwwzehr/research/database/), translated into amino acid sequences, aligned to the existing hidden Markov model alignment using the Quick Align function, and nucleic acids were realigned to the aligned amino acids in the ARB software environment. Sequences generated from the nutrient addition bioassays were clustered at 97% nucleotide similarity using CD-HIT-EST [45]. Nucleic acid trees used the Jukes-Candor correction for branch length. Trees generated in ARB were exported into iTOL for the display of associated metadata. All partial *nifH* sequences recovered were submitted to Genbank under Accession numbers KF515738 - KF515848.

## Results

### Hydrographic and nutrient profiles

**Figure 2:**
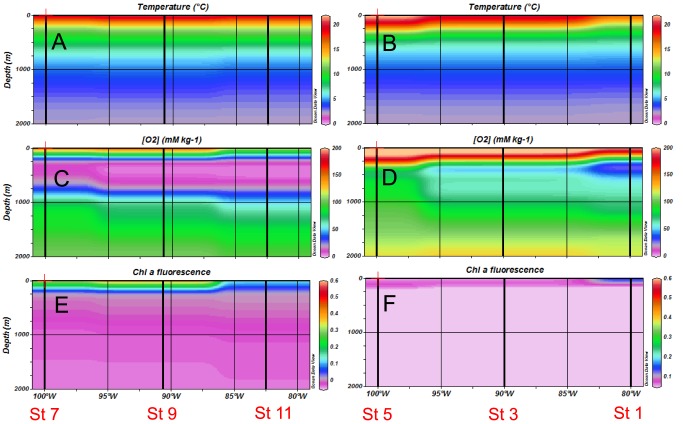
Horizontal and vertical distributions of hydrological and biogeochemical parameters during the 2010 cruise (R/V Atlantis). (A, B) temperature, (C, D) dissolved oxygen, (E, F) chlorophyll fluorescence in the northern transect (left panels) and southern transect (right panels).

**Figure 3:**
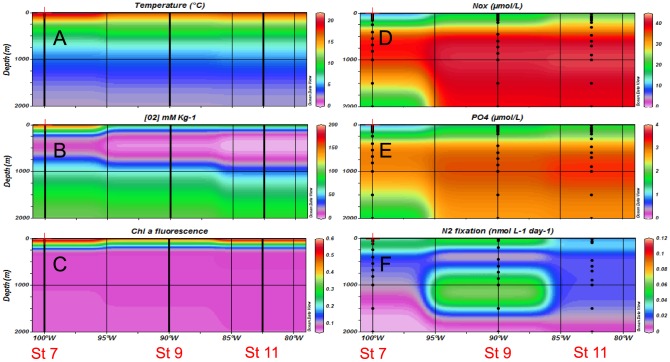
Horizontal and vertical distributions of hydrological and biogeochemical parameters during the 2011 cruise (R/V Melville) – Northern transect (10°S). (A) temperature, (B) dissolved oxygen, (C) chlorophyll a fluorescence, (D) NO_3_^−^ concentrations, (E) PO_4_^3−^ concentrations, (F) Mean N_2_ fixation rates (n = 3).

**Figure 4:**
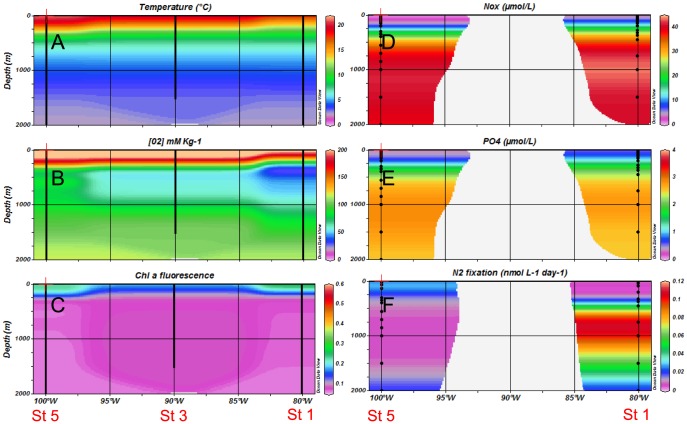
Horizontal and vertical distributions of hydrological and biogeochemical parameters during the 2011 cruise (R/V Melville) – Southern transect (20°S). (A) temperature, (B) dissolved oxygen, (C) chlorophyll a fluorescence, (D) NO_3_^−^ concentrations, (E) PO_4_^3−^ concentrations, (F) Mean N_2_ fixation rates (n = 3).

During both cruises, oceanographic conditions were consistent with active wind-driven upwelling off the coast of Northern Chile and Peru (Fig. 1), associated with a vertically and horizontally extensive OMZ (Fig. 2C and 3B). During the 2010 cruise (R/V Atlantis, El Niño), the zone of decreasing dissolved oxygen (oxycline) was located at ca. 70–100 m at stations 11 and 9, and suboxic conditions ([O_2_] <20 µmol kg^−1^ [46]) were reached at 125 m and 130 m respectively at these two stations. The suboxic zone expanded from 130–750 m at Station 11. During the 2011 cruise (Fig. 3B, R/V Melville, La Niña conditions), the oxycline was shallower (ca. 30–40 m) at stations 11 and 9 compared to 2010, and suboxic conditions were reached at 65 m at both stations. The suboxic zone expanded from 70–850 m at Station 11. On the southern transect (Stations 1, 3 and 5, Fig. 2D and 4B), the water column was well oxygenated during both cruises (ca. 200 µmol kg^−1^) over the first 200 m, and O_2_ concentrations decreased with depth to reach minimum values of ca. 80 µmol kg^−1^ at 300 m depth.

During the 2011 cruise, on the northern transect at Stations 7 and 9 (Fig. 3D), surface NO_x_ concentrations were ca. 6 µmol L^−1^, increased quickly with depth (nitracline ca. 50 m) to reach ca. 37 µmol L^−1^ in the core of the OMZ. Close to the coast (Station 11), the nitracline was shallower (ca. 25 m) than that of the oceanic stations, and NO_x_ concentrations increased quickly to reach concentrations of ca. 30–40 µmol L^−1^ in the core of the OMZ. On the southern transect (Fig. 4D), NO_x_ concentrations were ca. 0.10 µmol L^−1^ over the first 100 m of the water column. The depth of the nitracline was 85 m and 130 m at Stations 1 and 5, respectively, and concentrations increased progressively to reach values of ca. 40 µmol L^−1^ below the oxygen minimum and down to 2000 m. PO_4_ concentrations (Fig. 3E and 4E) followed the same trend as NO_x_ in the northern transect, with surface concentrations of 0.40–0.60 µmol L^−1^ and a shallower phosphocline near the coast (Station 11) compared to open ocean stations. In the southern transect, surface PO_4_ concentrations were lower compared to those of the northern transect (0.03–0.30 µmol L^−1^) and the phosphocline was located deeper (ca. 150 m).

During both cruises, chorophyll *a* fluorescence (Fig. 2E–F, 3C, 4C) was highest at stations located along the northern transect. It was much lower in 2010 compared to 2011, especially at stations nearest the coast of Peru on the Northern transect.

### Vertical profiles (0 to 2000 m) of N_2_ fixation

**Figure 5:**
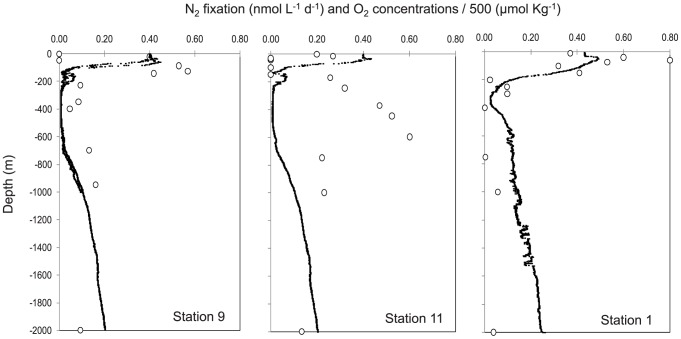
Vertical profiles (0–2000 m) of N_2_ fixation rates (nmol L^−1^ d^−1^) during the 2010 cruise (R/V Atlantis). Open circles: individual N_2_ fixation measurements at each depth. Black line: dissolved oxygen concentrations (µmol Kg^−1^) divided by 500 to fit on the same scale.

During the 2010 cruise, N_2_ fixation was detected in 34 of 40 samples representing all three stations (Fig. 5). The overall range of rates measured over the cruise was from the detection limit to 0.80 nmol L^−1^ d^−1^. The highest rates were measured in O_2_ deficient waters at the oxyclines or in the core of the OMZ (Fig. 5), and reached values up to 0.57 nmol L^−1^ d^−1^, 0.6 nmol L^−1^ d^−1^ and 0.53 nmol L^−1^ d^−1^ at Stations 1, 11 and 9, respectively. Below the OMZ, rates were always measurable and were at 1000 m depth 0.16 nmol L^−1^ d^−1^, 0.23 nmol L^−1^ d^−1^ and 0.06 nmol L^−1^ d^−1^ at these stations. Integrated rates over the 2000 m water column were 155 µmol N m^−2^ d^−1^ at Station 1, 288 µmol N m^−2^ d^−1^ at Station 9, and 509 µmol N m^−2^ d^−1^ at Station 11 (Table 1). The average integrated rate over the cruise was 317 µmol N m^−2^ d^−1^. Integrated N_2_ fixation rates in the aphotic zone accounted for 73 to 99% of the rates measured over the entire water column depending on the station. When considering all the stations, the average areal rate in the aphotic zone was 87% of the total rate over the entire water column (Table 1).

**Table 1:** Areal N_2_ fixation rates (µmol N m^−2^ d^−1^) calculated from measurements performed in the euphotic and aphotic zones during the 2010 and 2011 cruises.

Station	Total 0–2000 m (µmol N m^−2^ d^−1^)	Euphotic (µmol N m^−2^ d^−1^)	Aphotic (µmol N m^−2^ d^−1^)
**2010**			
Station 9	288	49	239
Station 11	509	8	501
Station 1	155	71	84
**Average**	**317**	**43 (13%)**	**275 (87%)**
**2011**			
Station 7	50±39	10±10	40±29
Station 9	101±79	09±04	92±74
Station 11	25±17	04±02	21±14
Station 5	24±14	05±05	19±09
Station 1	118±87	01±01	117±86
**Average**	**64**	**06 (10%)**	**58 (90%)**

Uncertainties are derived from the standard errors of triplicate measurements for the 2011 cruise.

**Figure 6:**
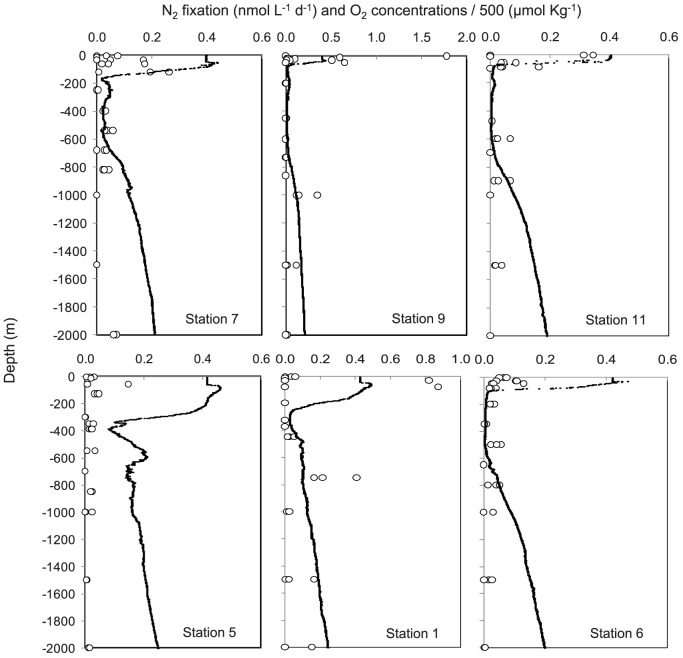
Vertical profiles (0–2000 m) of N_2_ fixation rates (nmol L^−1^ d^−1^) during the 2011 cruise (R/V Melville). Open circles: individual N_2_ fixation measurements at each depth. Black line: dissolved oxygen concentrations (µmol Kg^−1^) divided by 500 to fit on the same scale.

During the 2011 cruise, N_2_ fixation rates were significantly greater than zero in 140 of the 216 measurements made (Fig. 3F, Fig. 6). The overall range of rates measured was from detection limit to 0.26±0.12 nmol L^−1^ d^−1^. In the northern transect (Fig. 3F, Fig. 6), the highest rates of N_2_ fixation over the vertical profiles were measured in the oxycline as in 2010, and mean rates (n = 3) reached 0.15±0.13 nmol L^−1^ d^−1^ at Station 7 and 0.19±0.28 nmol L^−1^ d^−1^ at Station 9 at the oxycline. At station 11, the highest rates were found in surface waters (0.22±0.19 nmol L^−1^ d^−1^) but rates at the oxycline were also measurable (0.06±0.03 nmol L^−1^ d^−1^). Below the OMZ (ca. 400–2000 m), rates were also measurable and ranged from 0.00±0.01 to 0.21±0.13 nmol L^−1^ d^−1^, the highest rates being measured at station 9 at 1000 m depth. In the southern transect (Fig. 4F, Fig. 6), the rates ranged from 0.00±0.01 to 0.26±0.12 nmol L^−1^ d^−1^, the highest rates being observed at Station 1 just below the second oxycline at 750 m depth. At this station, aphotic rates were measurable at 450 m, 750 m, 1000 m and 1500 m depth. Integrated rates over the 2000 m water column ranged from 24±14 µmol m^−2^ d^−1^ at Station 5 to 118±87 µmol m^−2^ d^−1^ at Station 1 (Table 1). The average integrated rates over the 2011 cruise were 64 µmol m^−2^ d^−1^. Integrated N_2_ fixation rates over the aphotic zone accounted for 90% of total rates measured over the entire water column (Table 1) over the cruise.

### Nutrient addition bioassays in the core of the OMZ

**Figure 7:**
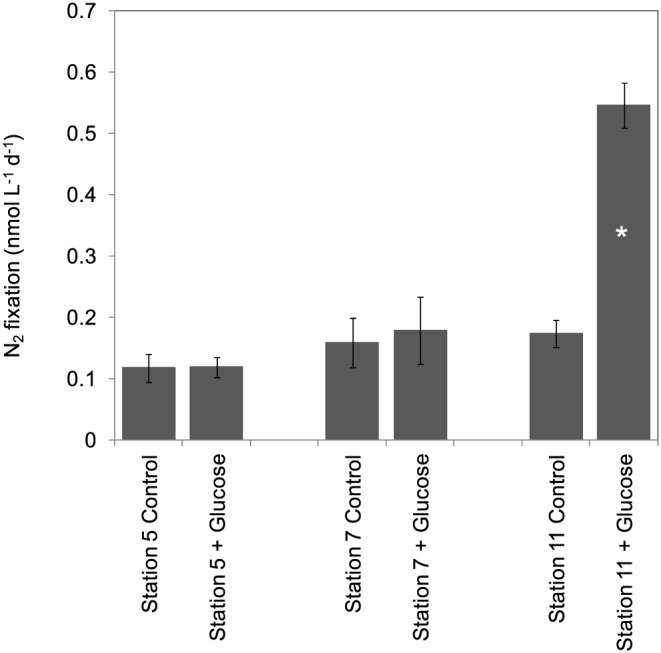
Effect of glucose additions on mean N_2_ fixation rates (n = 3) during the Atlantis cruise (2010). Data from bioassay experiments performed in the OMZ at Stations 5, 7 and 11. The error bars represent the standard deviation of triplicate incubations. Treatment means were compared using the 2-tailed non parametric Mann-Whitney mean comparison test (n = 3, α = 0.05, unpaired samples). Means that are significantly different (p<0.05) from the control are labeled with an asterisk.

During the 2010 cruise, nutrient concentrations in the core of the OMZ (140 to 450 m) where experiments were performed ranged from 24.0 to 37.0 µmol L^−1^ for NO_x_ and 1.20 and 3.00 µmol L^−1^ for PO_4_^3−^ (data not shown). Mean N_2_ fixation rates in control bottles at Stations 5, 7 and 11 were 0.12±0.02, 0.16±0.04 and 0.17±0.02 nmol N L^−1^ d^−1^, respectively (n = 3; Fig. 7). At the 2 most oceanic Stations 5 and 7, glucose amendments did not result in any significant increase of N_2_ fixation (p>0.05). At Station 11 near the Peruvian coast, glucose amendments resulted in a significant (p<0.05) increase in N_2_ fixation rates by a factor of 3.2, to reach 0.56±0.04 nmol N L^−1^ d^−1^ (Fig. 7).

**Figure 8:**
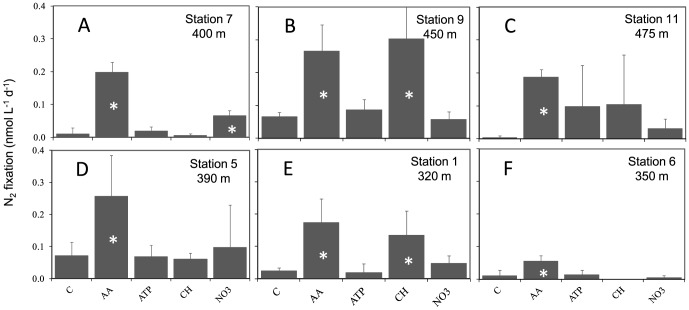
Effects of nutrient additions on mean N_2_ fixation rates (n = 3) during the Melville cruise (2011) (C: Control, AA: Amino acids, CH: Carbohydrates). Data from bioassay experiments performed in the OMZ at (A) Stations 7, (B) Station 9, (C) Station 11, (D) Station 5, (E) Station 1, and (F) Station 6 during the Melville cruise (2011). The error bars represent the standard deviation of triplicate incubations. Treatment means were compared using the 2-tailed non parametric Mann-Whitney mean comparison test (n = 3, α = 0.05, unpaired samples). Means that were significantly different (p<0.05) from the control are labeled with an asterisk.

During the 2011 cruise, nutrient concentrations in the core of the OMZ (320 to 475 m) where experiments were performed ranged between 33.12 and 38.72 µmol L^−1^ for NO_x_ and from 2.33 to 3.03 µmol L^−1^ for PO_4_. Mean N_2_ fixation rates in the control bottles ranged from 0.00±0.01 at Station 11 to 0.07±0.01 and 0.07±0.04 nmol N L^−1^ d^−1^ at Stations 9 and 5, respectively (n = 3; Fig. 8). At Stations 1 and 9, N_2_ fixation rates were significantly (p<0.05) stimulated by simple carbohydrate additions by a factor of 5.5 and 4.6, to reach 0.14±0.07 and 0.30±0.30 nmol N L^−1^ d^−1^, respectively (Fig. 8). At all stations, the addition of amino acids resulted in a significant (p<0.05) increase in N_2_ fixation rates, by a factor of 4 to 7. The highest rates were reached at Station 9 after AA additions with 0.27±0.08 nmol N L^−1^ d^−1^. ATP addition never resulted in any significant increase of N_2_ fixation rates (p>0.05) and NO_3_^−^ additions never resulted in any decrease of N_2_ fixation rates (p>0.05). However, at Station 7, NO_3_^−^ additions resulted in a significant (p<0.05) increase of N_2_ fixation by a factor of 7 to 0.07±0.02 nmol N L^−1^ d^−1^.

### Phylogenetic characterization of diazotrophs in 2010 glucose addition bioassays

**Figure 9:**
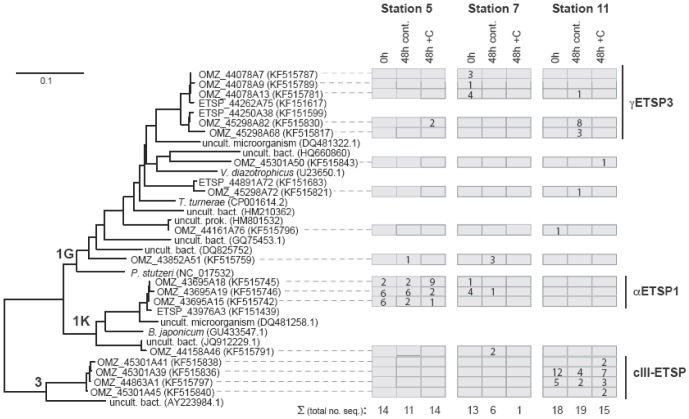
Results from the molecular analyses of the 2010 glucose amendment experiments. Neighbor joining tree of partial *nifH* nucleotide sequences. Nodes are labeled with *nifH* cluster designations according to the convention established [74]. The number of sequences recovered from stations and treatments for each phylotype are indicated in boxes to the right of the tree.

A full phylogenetic characterization of diazotrophs in the upper 200 m of the ETSP water column was performed during the same cruises and is detailed in a companion paper [42]. In this study we report the complementary phylogenetic characterization of samples from the core of the OMZ (Fig. 8). Partial *nifH* sequences recovered during deep glucose addition bioassays during 2010 at Stations 5, 7 and 11, indicated that diazotrophs were present in the deep waters of the OMZ. The diazotrophic community was comprised of non-cyanobacterial diazotrophs affiliated with *nifH* clusters 1K (predominantly comprised of α-proteobacteria), 1G (predominantly comprised of γ-proteobacteria), and 3 (sulfate reducing genera of the δ-proteobacteria as well *Clostridium* spp., *Vibrio* spp, etc.) (Fig. 9). Clear differences exist between OMZ diazotrophic community composition at each station. The Station 5 community was dominated by *nifH* cluster 1K sequences, many of which are closely related to a phylotype (94–97% nucleic acid similarity) originally reported at Hydrostation S (North Atlantic) from a depth of 1000 m (BT5167A10 (DQ481253) [47]), although a few putative γ-proteobacteral (1G) sequences were also recovered that affiliated with γETSP3, a cluster recovered from the ETSP [42]. Although the lowest number of total sequences was recovered from Station 7, they were mainly affiliated with cluster 1G, along with a few 1K sequences. In contrast, clone libraries from Station 11 were dominated by cluster 3 sequences, along with a few 1G sequences, but no 1K sequences (Fig. 9).

Despite being prevalent in clone libraries, both αETSP1 and cIII-ETSP groups were not detected at abundances great enough to quantify using Taqman® qPCR assays during the course of these experiments (see Figuer S1 in File S1). Because the abundances of these targets did not increase as a result of nutrient amendments, it is difficult to speculate whether any of them were responsible for the increased N_2_ fixation rates we measured after glucose addition.

## Discussion

### Active N_2_ fixation in deep and NO_3_-rich waters of the ETSP

In this study, we measured during 2 consecutive years N_2_ fixation in surface waters affected by the OMZ, but reveal that N_2_ fixation below the euphotic zone is more important: 87 and 90% of total areal N_2_ fixation were measured in the aphotic zone in 2010 and 2011, respectively. In these aphotic layers, volumetric N_2_ fixation rates were relatively low (<1.00 nmol N L^−1^ d^−1^), but when integrated over the whole aphotic layer, they ranged from 84 to 501 µmol N m^−2^ d^−1^ in 2010 and from 19±09 to 117±86 µmol N m^−2^ d^−1^ in 2011 (Table 1). In 2011, rate measurements were replicated (triplicates) and calculations performed very carefully using a real T0 for every depth. These 2011 measurements are thus more reliable than those measured in 2010. These measurements in aphotic waters add new information compared previously published studies [26], [28] in the area. The hypotheses explaining the persistence of N_2_ fixation in these high NO_x_ (ca. 40 µmol L^−1^) environments are largely developed in the companion paper [28]. First, fixed N loss processes occur in this region [31], [48], creating a deficit of N relative to P, which is potentially favorable for N_2_ fixation [24]. In particular, anammox removes NH_4_^+^, which has an immediate inhibitory effect on N_2_ fixation [49]. Secondly, N_2_ fixation is an anaerobic process [50] due to the irreversible inactivation of the nitrogenase enzyme by O_2_ [51]. It is possible that the low O_2_ concentrations in the OMZ and down to 2000 m contribute to the protection of nitrogenase [52], decrease the energy cost of maintaining intracellular anaerobiosis [53], and thus facilitate N_2_ fixation. Finally, redox conditions in the OMZ favor the equilibrium formation of the most bioavailable form of iron Fe^2+^ [54], which could help to support the high Fe requirements of nitrogenase [16], [17]. For these reasons, OMZs and deeper waters may represent favorable ecological niches for N_2_ fixation, as shown in this study.

### Potential impacts on N budgets in the ETSP

Aphotic N_2_ fixation is currently ignored in oceanic N budgets based on biogeochemical rate measurements. However, this dataset indicates that rates in aphotic waters of the ETSP are of the same order of magnitude than those commonly measured in the tropical and sub-tropical NO_3_^−^-depleted surface ocean (Table 2), where N_2_ fixation has commonly been studied. The potential significance of the N_2_ fixation rates measured in our study can be evaluated by comparing them with fixed N losses via denitrification and anammox measured in the same region. N losses in the ETSP have been estimated to range from 9 to 25 Tg N yr^−1^ (Table 3, [31], [55], [56], [57]) in the upwelling area extending 175 km offshore, and 1860 km along the Peruvian-Chilean coast with an area extant of 3.26×10^11^ m^2^ [31], [56]. If we consider the same spatial extent for N_2_ fixation, this process could potentially add 0.04 to 0.9 Tg N yr^−1^ (Table 3), counterbalancing 0.16 to 10% of the estimated N loss processes in this area (calculations have been performed only using numbers from the 2011). However, the anammox and denitrification measurements mentioned above [31] were performed under conditions of excess substrate availability and therefore represent maximum estimates of N loss rates. In contrast, the ^15^N_2_ bubble method used to quantify N_2_ fixation [58] may underestimate rates [38], [39].

**Table 2:** Examples of published studies showing the range of oceanic N_2_ fixation areal rates measured in some contrasting oceanic environments.

Location	Areal rates (µmol m^−2^ d^−1^)	Integration depth (m)	Reference
Hypoxic basin (Southern California Bight)	150	885	[37]
ETSP coastal OMZ	7–190	120	[26]
ETSP	0–148	150–200	[28]
ETSP subtropical gyre	12–190	150–200	[14]
Eastern North Pacific gyre	520	mixed layer	[20]
North Atlantic	59–898	15 (*Trichodesmium* bloom)	[3]
*ETSP aphotic zone*	*19–501*	*2000*	*This study*

**Table 3:** Comparison between estimated fixed N losses via denitrification and anammox and fixed N gains via N_2_ fixation (estimated from the 0–2000 m depth integrated rates measured in this study) in the ETSP.

Area considered	N losses	N gains (based on 2011 cruise)	N gains (based on 2010+2011 cruise)
(m^2^)	(Tg.yr^−1^)	(Tg.yr^−1^)	(Tg.yr^−1^)
*3.26×10^11^	**9–25	0.04–0.9	0.05–1.1
2.23×10^12^	9–25	0.3–1	0.3–7

[31], [56]. Upwelling area extending 175 km from, and 1860 km along the Peruvian-Chilean coast

[31], [55], [56], [57]. Estimates from

Secondly, denitrification and anammox are restricted to subsurface suboxic or anoxic waters [59], whereas N_2_ fixation is not. Further, denitrification and anammox appear to be restricted to the coastal upwelling system within ca. 175 km of the Peruvian-Chilean coast (the few data available at open ocean stations indicate that N loss processes were below detection limit during the 2010 cruise, Hamersley et al., (Pers. Com.)). N_2_ fixation in the ETSP is active over a much greater spatial extent than N loss processes. If we consider the spatial extent of the N_2_ fixation measurements in the ETSP covered by our cruises (2.23×10^12^ m^2^), we estimate that N_2_ fixation could potentially add 0.3 to 1 Tg N to the system in this area and therefore could compensate for up to 11% of the estimated N loss processes in the upwelling region of the ETSP (Table 3) (without taking into account methodological under- or overestimations). These estimates of N gains are the minimum ones calculated by taking into account only the 2011 cruise. If we take into account the 2010 cruise, N_2_ fixation could potentially compensate up to 0.3 to 7 Tg N to the system (Table 3) (i.e. up to 78% of N losses). N_2_ fixation in deep waters of the ETSP may be a significant source of N for the ETSP, and needs to be taken into account in future N budgets. Further coupled measurements between N gain and loss processes at the same stations/depths need to be performed to better constrain the magnitude of N gains in this region.

### Effects of nutrients on N_2_ fixation

The diazotrophic community of the ETSP characterized in this study, as well as in a companion study [42] is comprised of an assemblage of non-cyanobacterial diazotrophs, and little can be inferred about their metabolism from partial *nifH* sequences. However, we performed nutrient addition bioassays using molecules representing common labile components of the dissolved organic matter pool in marine waters (simple carbohydrates, amino-acids and ATP), which shed some light on nutrient control of N_2_ fixation in the core of the OMZ. Our results indicated that simple carbohydrate additions significantly stimulated N_2_ fixation at stations located nearest the coast during both cruises and at Station 9 during the 2011 cruise (Figs. 7, 8). In OMZs, organic C is largely supplied by vertical flux of planktonic production from shallower layers or by horizontal transport [60]. Thus this supply of organic C is not constant but rather episodic, which could explain why N_2_ fixation appears so variable in space, in time, and between cruises and years, as reported in the present study and by [26]. This seems to be the case for N loss processes as well, since organic C supply has been correlated with regional denitrification [60] and anammox [31], [61] rates in OMZs. Ward et al. [62] demonstrated that denitrification rates were significantly stimulated in the OMZ of the ETSP by organic C additions. To our knowledge our study is the first designed to study the response of diazotrophs to nutrient additions in the OMZ. In surface waters, significant stimulation of N_2_ fixation rates by glucose additions have been reported during the same cruise at Station 9 [28]. A significant stimulation of bacterial production after glucose amendments in surface waters of the Chilean upwelling system have also been reported [63]. Finally, in surface waters of the southwest Pacific [64], reported a significant increase of *nifH* gene copies of unicellular diazotrophic cyanobacteria such as Group A (UCYN-A) and *Crocosphaera* after glucose and mannitol additions, hypothesizing that this capacity may allow conservation of energy by rapid uptake and recycling of sugars. However, it has to be noted that the large variability in the response to carbohydrates addition (high standard deviation at Station 11 for example) could be explained by the fact that it may be coincidental whether the taxa that benefit from the enrichment possess the nifH gene.

Because the organic C molecules tested here are also energy-rich molecules easily entering catabolic pathways, one could interpret our results to be indicative of limitation either by energy or by assimilative C availability. However, in our experiments N_2_ fixation was not stimulated by ATP additions at any station, indicating that C and not energy might have been the proximate limiting factor. In some oligotrophic P-limited environments, ATP is also a source of P for bacteria and uptake rates of ATP exceed those of glucose [65]; however, in OMZs, P is not limiting relative to N, which may further restrict the ability of ATP to stimulate N_2_ fixation rates in our bioassays. In contrast, the addition of free amino acids stimulated N_2_ fixation at all stations tested; this has also been shown in aphotic oxynenated waters of the Red Sea [66]. Amino acids are a source of both C and N, and it has been suggested that it is energetically advantageous for microbes to use preformed compounds such as amino acids rather than glucose as C sources [67]. In terrestrial legume-rhizobium symbioses, the diazotrophic bacteria assimilate amino acids such as glutamic acid provided by the host, which facilitate both dicarboxylate oxidation and ammonium assimilation into asparagine [68]. In *Azospirillum sp.*, additions of glutamic acid also stimulated N_2_ fixation activity [69] by providing a C and energy source to the diazotrophs, while N was still provided via N_2_ fixation. It may be that similar nutrient assimilation dynamics are occurring in diazotrophs in the ETSP OMZ.

Ambient NO_x_ concentrations were high (ca. 30–40 µmol L^−1^) at all stations where nutrient additions were performed, and NO_3_^−^ additions (8 µmol L^−1^) never resulted in N_2_ fixation inhibition at any station. As the metabolic potential of diazotrophs present in the OMZ have not yet been fully characterized, we do not know if they possess genes for reduction and assimilation of NO_3_^−^ or NO_2_^−^. Detailed studies at the single cell level would be needed to characterize the metabolism of these organisms and understand why microbes fix N_2_ in the presence of so much NO_3_^−^. In addition to possible energy and C, N and P sources derived from molecules like amino acids, carbohydrates or ATP, electron sources and donors are also very important to know for characterizing the physiology of the diazotrophs present in the ETSP. Molecules like O_2_, NO_3_^−^ and less favorably SO_4_^2−^ are common electron acceptor and they are used for different types of respirations like aerobic respiration, or anaerobic denitrification and sulfate reduction. These different respiratory pathways potentially supporting N_2_ fixation are performed by organisms with different physiology which each have their own environmental sensitivities for fixing N_2_.

### Phylogenetic characterization of diazotrophs

Our characterization of the diazotrophic community in the core of the OMZ revealed the presence of potential N_2_-fixing heterotrophs based on the presence of the *nifH* gene. We did not detect the cyanobacterial diazotrophs commonly found in other regions of the open ocean; in contrast, most of the *nifH* genes amplified from the OMZ clustered with α-, γ- and δ-proteobacteria. This result is consistent with the observations of Turk-Kubo [42] in the upper 200 m of the ETSP water column, where 96% of sequences were also affiliated with proteobacteria. Based on these results, and other studies conducted in the ETSP and the South Pacific Gyre [14], [26], [70], it is clear that the ETSP diazotrophic community is different from other well-studied tropical and sub-tropical oceans such as that of the North Pacific, North Atlantic and Indian Oceans. The cyanobacterial diazotrophic phylotypes commonly found at high abundances in these other ocean provinces appear to be either sporadically present at low abundances (i.e. *Trichodesmium*, UCYN-A), or undetected altogether (i.e. UCYN-B, diatom-diazotroph associations) in the ETSP.

The amplification of diverse non-cyanobacterial *nifH*-containing organisms from OMZ waters in the ETSP affiliated with *nifH* clusters 1K, 1G and 3, is consistent with the findings of other studies conducted in anaerobic waters [26], [36], [71] and in abyssopelagic waters [47]. However, the results from this study underscore the difficulty inherent in identifying the diazotrophic community responsible for N_2_ fixation rates. It is important to note that *nifH* cluster 1K sequences have been reported as contaminants in many studies, including a study in the ETSP [42]. However, none of the sequences recovered here had greater than 90% amino acid similarity and 83% nucleic acid similarity to reported contaminants. Nevertheless, as a result of the use of highly degenerate primers and nested PCR cycles necessary to amplify this important but low-abundance gene target, contamination must always be considered as a source for heterotrophic diazotroph sequences, whether from PCR and DNA extraction reagents or from sampling or handling procedures, despite the screening of PCR and reagent blank controls as in this study.

Furthermore, although it is clear that a diverse assemblage of non-cyanobacterial *nifH*-containing organisms are present in the OMZ of the ETSP, the best methodologies currently available to characterize dominant members of the diazotrophic community (PCR amplification using degenerate *nifH* primers) often identify organisms present at extremely low levels when targeted using quantitative approaches (i.e. qPCR; Fig. S1 in File S1) [42], [47], [71]. This, in turn, makes it difficult to argue that these organisms are capable of fixing N_2_ at cell-specific rates great enough to account for measured bulk rates. An analysis of the expected N_2_ fixation rates based on abundances and plausible cell-specific N_2_ fixation rates in the ETSP discussed in [42] indicate that these proteobacteria are unlikely to be responsible for all the measured bulk rates and therefore other N_2_-fixing organisms could be responsible for a part of N_2_ fixation in this region but may remain uncharacterized. Identifying which organisms are actively transcribing *nifH* using techniques such as reverse transcription (RT)-qPCR might provide more insight into which diazotrophic taxa are actively fixing nitrogen. However, the challenge of identifying which organisms are important N_2_-fixers remains the same when designing qPCR primers from sequences derived from RT-PCR based clone libraries, and are further convoluted by potentially low transcript abundances per cell and/or the timing of sampling with respect to diel changes in *nifH* expression (even in the case of heterotrophs).

## Conclusions

This study provides one of the first estimates of N_2_ fixation rates in aphotic waters of the ETSP. It reveals that N_2_ fixation in aphotic environments is the largest contributor to total areal N_2_ fixation in ETSP. N_2_ fixation in high [NO_3_^−^] environments remains an enigma as it requires an additional energetic cost relative to NO_3_^−^ or NH_4_^+^. Further physiological studies are needed to understand the physiological regulation of N_2_ fixation, especially on newly discovered diazotrophic organisms. Contrary to N_2_ fixation performed in euphotic layer which sustains new primary production [3], aphotic N_2_ fixation may sustain organic matter remineralization. These new sources of N could potentially compensate for as much as 78% of the estimated N loss processes in ETSP, indicating that they need to be taken into account in marine N budgets. Phylogenetic studies confirm the presence of diazotrophs in the deep waters on the OMZ, which are distinct from cyanobacterial phylotypes commonly found in surface oligotrophic waters of the tropical ocean. Organic and inorganic nutrient addition bioassays reveal that amino acids and simple carbohydrates stimulate N_2_ fixation in the core of the OMZ, and the episodic supply of these nutrients from upper layers may explain the large temporal and spatial variability of N_2_ fixation in the ETSP. Research on marine heterotrophic N_2_ fixation is at its beginning and significant progress needs to be made in the refinement of the methods to estimate planktonic N_2_ fixation in OMZs (^15^N_2_ bubble method versus ^15^N_2_-enriched seawater) from bulk measurements to single cells analysis. The ^15^N-enriched seawater method should be coupled to oxygen-free and trace metal-clean procedures to provide more accurate estimates. Progress also needs to be made in the characterization of the community responsible for N_2_ fixation in these deep waters, as well as the control of their population dynamics by the supply of organic matter. Estimates of global N_2_ fixation based on field measurements [5], [72] are presently lower than geochemically-based (nutrient stoichiometry and isotopic ratio) estimates [73]. Taking into account deep N_2_ fixation might help to resolve some of this discrepancy. However, progress also needs to be made in the quantification of N loss processes, as recent studies indicate that they may be less sensitive to oxygen than previously thought [59], further complicating the N budget in the ETSP. In future studies, N gain and loss measurements need to be coupled in space and time to further resolve the N budget in the ETSP.

## Supporting Information

File S1 **Supporting methods, Table S1, and Figure S1.**
(DOCX)
