# Supplementary material for: Aphotic N2 Fixation in the Eastern Tropical South Pacific Ocean
Source: PLoS One. 2013 Dec 12;8(12):e81265. doi: 10.1371/journal.pone.0081265 (PMC3861260; doi:10.1371/journal.pone.0081265)
Supplement: File S1 — Supporting methods, Table S1, and Figure S1. (DOCX) [file pone.0081265.s001.docx]

**Supporting Methods**

*Quantitative characterization of diazotrophs*

Two new Taqman qPCR assays were designed using Primer3Plus [[75](#_ENREF_46)] based on *nifH* sequences dominant in clone libraries recovered from the bioassays. αETSP1 targets a cluster of *nifH* 1K sequences (putative α-proteobacteria) and cIII-ETSP targets *nifH* cluster III sequences (Table S1). A third qPCR assay targeting a subset of the *nifH* 1G cluster sequences, originally described in [[42](#_ENREF_30)], was also utilized (Table S1). All qPCR primers and probes were synthesized by Sigma Oligos (Sigma-Aldrich, St. Loius, MO). Generation of recombinant plasmid standards, qPCR reaction components and thermocycling conditions, as well as calculation of the concentration of *nifH* gene copies L^-1^followed the approach described in [76]. The limit of detection (LOD) and limit of quantitation (LOQ) for these samples was 12 and 200 *nifH* gene copies reaction^-1^ (or 6 and 50 *nifH* gene copies L^-1^), respectively.

All qPCR preparations were performed in a PCR-amplicon free facility at UCSC described in [42].

**Table S1.**

|  |  |  |  |  |  |
| --- | --- | --- | --- | --- | --- |
|  |  | **oligonuclotide sequence (5' - 3')** | | |  |
| **qPCR assay target** | **Sequence ID and Accession # of target** | **Forward primer** | **Probe (5'-FAM, 3'-TAMRA)** | **Reverse primer** | **Reference** |
| γETSP3 | ETSP_44879A48 (KF151661) | TCA TGG AAA TGG CTG CTG AAG | GGG CTA CGG CGA CAT CAA GTG CG | GAT TAC ACC GCG ACC AGC AC | [30] |
| αETSP1 | ETSP_OMZ_43695A19 (KF515746) | TCG AGG ACG TGA TGA A | TCA TCA CCT CGA TCA ACT TCC TCG A | GTA GGA CAC ATA GTC GA | This Study |
| cIII-ETSP | ETSP_OMZ_45301A39 (KF515836) | GGA AGC CGC TGC GTG GAA TC | ACC GGG CGT CGG ATG TGC CGG TCG GGG | TCA TAG GCA CCC AGC TGT TCG | This Study |
|  |  |  |  |  |  |

**Legends**

**Table S1.** Description of qPCR assays targeting non-cyanobacterial diazotrophs utilized in the analysis of 2010 glucose addition bioassays.

**Figure S1.** Results from the molecular analyses of the 2010 glucose amendment experiments.

Abundances (*nifH* copies L^-1^) of phylotypes γETSP3 (A), αETSP1 (B) and cIII-ETSP (C) in each of the three experiments, determined using qPCR. Detection limit and limit of quantification are represented by dotted and solid horizontal lines, respectively.  Error bars are derived from treatment replicates.

**References cited.**

75. Untergasser A, Cutcutache I, Koressaar T, Ye J, Faircloth BC, et al. (2012) Primer3—new capabilities and interfaces. Nucleic Acids Research 40: e115-e115.

76. Goebel NL, Turk KA, Achilles KM, Pearl RW, Hewson I, et al. (2010) Abundance and distribution of major groups of diazotrophic cyanobacteria and their potential contribution to N2 fixation in the tropical Atlantic Ocean. Environmental Microbiology 12: 3272–3289.
